# Health Information on Internet: Quality, Importance, and Popularity of Persian Health Websites

**DOI:** 10.5812/ircmj.12866

**Published:** 2014-04-05

**Authors:** Mahnaz Samadbeik, Maryam Ahmadi, Ali Mohammadi, Beniamin Mohseni Saravi

**Affiliations:** 1 Department of Health Information Technology, Lorestan University of Medical Sciences, Khorramabad, IR Iran; 2 Department of Health Information Management, School of Health Management and Information Science, Iran University of Medical Sciences, Tehran, IR Iran; 3 Health Management and Economics Research Center, School of Health Management and Information Sciences, Iran University of Medical Sciences, Tehran, Iran; 4 Treatment Vice Chancellor Medical Record Department, Mazandaran University of Medical Sciences, Sari, IR Iran

**Keywords:** Evaluation Studies, Internet, Health

## Abstract

**Background:**

The Internet has provided great opportunities for disseminating both accurate and inaccurate health information. Therefore, the quality of information is considered as a widespread concern affecting the human life. Despite the increasingly substantial growth in the number of users, Persian health websites and the proportion of internet-using patients, little is known about the quality of Persian medical and health websites.

**Objectives:**

The current study aimed to first assess the quality, popularity and importance of websites providing Persian health-related information, and second to evaluate the correlation of the popularity and importance ranking with quality score on the Internet.

**Materials and Methods:**

The sample websites were identified by entering the health-related keywords into four most popular search engines of Iranian users based on the Alexa ranking at the time of study. Each selected website was assessed using three qualified tools including the Bomba and Land Index, Google PageRank and the Alexa ranking.

**Results:**

The evaluated sites characteristics (ownership structure, database, scope and objective) really did not have an effect on the Alexa traffic global rank, Alexa traffic rank in Iran, Google PageRank and Bomba total score. Most websites (78.9 percent, n = 56) were in the moderate category (8 ≤ x ≤ 11.99) based on their quality levels. There was no statistically significant association between Google PageRank with Bomba index variables and Alexa traffic global rank (P > 0.05).

**Conclusions:**

The Persian health websites had better Bomba quality scores in availability and usability guidelines as compared to other guidelines. The Google PageRank did not properly reflect the real quality of evaluated websites and Internet users seeking online health information should not merely rely on it for any kind of prejudgment regarding Persian health websites. However, they can use Iran Alexa rank as a primary filtering tool of these websites. Therefore, designing search engines dedicated to explore accredited Persian health-related Web sites can be an effective method to access high-quality Persian health websites.

## 1. Background

Access to health information is fundamental to better health and has many benefits for patients and their families. This information increases knowledge about diseases and their control, enhances disease management and reduces patients’ anxiety, as well as encouraging them to more actively participate in care, make better informed medical decisions and have better acceptance of medical advices (1). The numerous advantages of the Internet, such as easy accessibility and mutual communication, made it a new and free source of disseminating health information, moving toward an information revolution (2-5). As a result, medical information that was previously hard to access is now broadly available to many people. So, this online information can bring a positive transformation in the health services provider and consumer relationship. Moreover, it prevents arbitrary decision-making by physicians and increases patients' responsibility for their own health (6, 7). Meanwhile, due to the accessibility to this ubiquitous medium, lay patients can complement the information obtained from physicians and easily learn what they need to know about prevention, diagnosis and treatment of diseases (8-10). Increase in the number of online health information consumers (10), portals (7) and websites shows that patients like to participate in health care and health decision-making (6, 11).

In the present era, generation and distribution of information has surpassed the human power in processing information and the information overloading problem has occurred. Due to the variety of health related websites, possibility of disseminating information by unspecialized persons and lack of a simple instrument for precise assessment and quality control of health websites, the quality of information is considered as a widespread concern affecting the human life (2, 6, 12-14). This concern is prompted by incomplete, inaccurate, misleading, out of date and biased information on health websites that have adverse impacts on patients and health care specialists and cause their failure in proper use of internet resources (4). In addition, health websites can also cause other problems such as dishonest advertising of unhealthy and dangerous products, jeopardizing healthcare provision, inappropriate use of users’ personal data and false online consultation (8, 15).

Nowadays in Iran, the Internet has had an increasingly significant growth in the number of users, Persian health websites and the proportion of Internet-using patients. For example, the number of internet users in Iran increased to 36500000 over 2011 which is more than a half of the total number of internet users in the Middle East (16, 17). Furthermore, some issues such as patients’ tendency to search for information on the Internet in their native language, the variable quality of online health information (2-4, 8, 10, 12, 18-20) and the absence of supervision on Persian health websites propose a necessity for quality evaluation of these websites. Although, only few studies have been performed to assess the quality of Persian medical and health websites (19, 20).

## 2. Objectives

The current study was conducted to assess the quality, popularity and importance of websites providing health-related information in Persian language.

## 3. Materials and Methods

### 3.1. Website Selection

**Figure 1.**
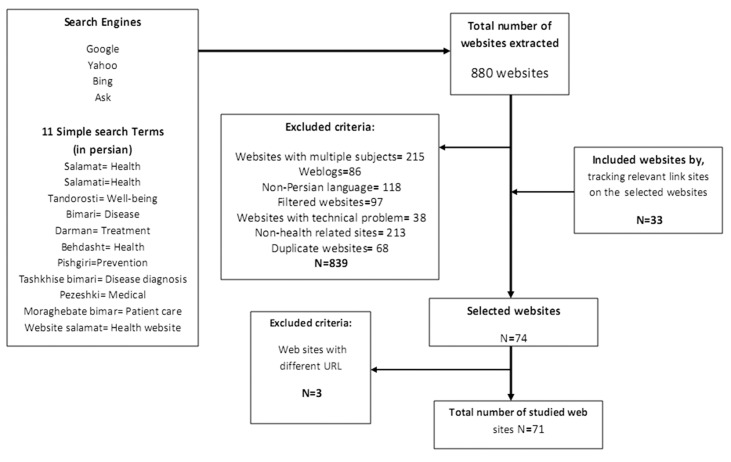
Flow of Websites Selection

Websites were identified by entering health-related keywords extracted from Persian medical terminology (21) into four most popular search engines of Iranian users based on the Alexa ranking at the time of study: Google (1), Yahoo (2), Bing (46) and MSN (59) (22). Between the first of November and the 13th of December 2011, these search engines looked for websites in Persian that contained the retrieved keywords. Analyzed websites were limited to the sites listed in the first two pages (20 sites) of the search engines result, because most web users use search engines as the primary method to find websites and are not expected to go beyond two pages when looking for information (23-25). At first, a list of 880 websites was generated. In the next step, by refining our search results 215 websites with multiple subjects, 86 weblogs, 118 websites in non-Persian language, 97 filtered websites, 38 websites with presented technical problem after multiple attempts at the time of study, 213 non-health related sites and 68 duplicate websites were excluded. Then, 33 websites were included by tracking relevant link sites on the selected websites. Moreover, 3 websites with multiple different URL addresses were recognized and excluded. Finally, 71 sites were identified to be completely investigated and analyzed (Figure 1).

### 3.2. Websites Characteristics

The websites were rated based on a range of properties including ownership structure, database, scope and objective (Table 1). The type of database was extracted from Domain Tools recognized as a leading website in domain research and monitoring (26). Moreover, the objective of website was found in the website goal statement.

**Table 1.** Site Characteristics Groups Based on the Ownership Structure, Database, Scope and Object

Site Characteristic	Results, %
**Site ownership**	
Government	15.9
Individual	49.3
Corporation	34.8
**Database**	
MS-SQL	46
My-SQL	54
**Scope**	
Specific	44.4
General	55.6
**Objective**	
Health promotion	57.9
Service delivery	8.8
Improve physician-patient relationship	1.8
Introduce individual	3.5
Common (multi object websites)	28.1

### 3.3. Websites Assessment

Website assessment was performed by the following qualified tools:

The Bomba and Land Index (version 2)

The Bomba and Land Consumer Health Rating Index (version 1) was developed specifically to evaluate websites targeted at health consumers based on the work of Slack and the HON code principles. It uses a series of guidelines with multiple subitems to score individual sites (7). The second version of this index was prepared after reviewing and ensuring its internal validity by Sullivan’s five step process (14). Version 2 of this quality index was chosen because of its specific characteristics, such as covering all the major dimensions of quality, weighting and adding the scores of multiple rating scales, having a rating scale giving more weight to content, usability, reliability and transparency over more technical features, being manageable (easy to use), being more transparent regarding the process of the instrument construction and validation, confirmed validity and reliability, and being dedicated to the health domain (4, 27). The guidelines of health quality index include:

Content: This guideline is performed in a 10-point scale and 6 weighted multipliers. It refers to whether the content of the website is medically sound, justifiable, clearly attributed, and subject to formal study. It also asks questions pertaining to the credentials of the content providers, whether there is a balanced presentation of the material and transparency of the site responsible person.

Usability: this guideline is performed in a 13-point scale and 5 weighted multipliers. It refers to clarity of language, navigation, and whether the user has the opportunity to post and ask questions, as well as obtaining online help when needed.

Fast, reliable and readily available: this guideline is performed in a 4-point scale and 4 weighted multipliers. It refers to whether the site is useable and loadable without any trouble.

Advertising and editorial policy and transparency of authorship and sponsorship: This guideline is performed in a 5-point scale and 3 weighted multipliers. It refers to whether all relevant contact, financial and support interests are declared.

Complimentary and interactive: This guideline is performed in a 6-point scale and 2 weighted multipliers. It refers to whether the site encourages contact with a health professional and offers links to such resources.

Confidentiality: This guideline is performed in a 7-point scale and 1 weighted multiplier. It refers to whether users' information is protected if patient data is collected.

Final total score: scores were calculated by summing the total number of positive responses, dividing by the total number of applicable subitems for that particular guideline, and multiplying by the weighted constant for that category. The maximum score of the index was 21 (6, 14, 28).

The content validity of this tool was confirmed by a panel of four subject matter experts and the same interpretation was reached for guidelines and subitems. In addition, for more accurate evaluation of the websites, regarding the scoring of two-part subitem questions, a half score (0.5) was assigned if the answer of one component was positive. Furthermore, a full score was given only to the subitem if the answers to both components were positive (including, subitem 4 of guidelines content, subitems 10 and 11 of usability guidelines, including, subitem 4 of availability guidelines, subitems 3 of advertising guidelines and subitems 4 of complimentary guidelines).

Due to the dynamic and variable features of the websites, the evaluation of the websites was separately performed by two raters using the Bomba index on the same day. An agreement and interrater reliability analysis using the Kappa statistic was performed to determine consistency among raters. There was a perfect interrater reliability based on the Kappa interpretation list prepared by Landis & Koch (29) (kappa = 0.89, P < 0.001). If the ratings of the two raters differed, a third rater (consensus) resolved any disagreements and a common score was agreed upon.

In this study, we used the 512 Kbps ADSL Internet service connection through Pars Online Internet Service Provider (ISP) by a wireless Meganet ADSL modem. The subitem 2 of availability guideline, namely "Did it take less than 8 seconds to download the homepage?", was answered by using two online automated tools including Self SEO (30) and iWEBTOOL (31). If the homepage load time took more than 8 seconds, the website was repeatedly evaluated by other online tools, encompassing Pingdom (32) and WebToolHub (33). The answer would be negative if the load time took more than 8 seconds using 3 or 4 tools. The first part of the subitem 4 of availability guideline, namely "Is each page useable (i.e. no broken links, images load, no pop-ups)?", was answered by using Broken Link Checker of the Axandra automated tool (34). The second part of the subitem 4 of availability guideline, namely “Can the site be viewed with another browser?", was answered based on three most popular browsers in Iran, Firefox, Internet Explorer and Chrome, from Aug 2011 to Aug 2012 , respectively (35). The answer of subitem 5 of confidentiality guideline, namely “Can a user edit their own information held by the site?", was obtained after registering on the assessed website. The quality level of each category was determined by the total Bomba score calculated (0-25) for each website. Table 2 shows the Bomba score and the corresponding quality level.

**Table 2.** Bomba Total Score and Quality Levels

Final Total Score (x)	Quality Level
**0 ≤ x ≤ 3.99**	very poor
**4 ≤ x ≤ 7.99**	poor
**8 ≤ x ≤ 11.99**	moderate
**12 ≤ x ≤ 15.99**	good
**16 ≤ x ≤ 21**	excellent

### 3.4. Google PageRank

Google PageRank is one of the methods Google uses to determine the relevance or importance of a page. The PageRank values range from 0 to 10, with higher values indicating greater importance (10). Using the Google Toolbars, the integer value (range 0 to 10) on the toolbar was recorded for all home pages of the selected websites on June 22, 2012.

### 3.5. Alexa Traffic Rank

Alexa traffic rank determines the websites popularity. It is based on three months of aggregated historical traffic data from millions of Alexa Toolbar users and data obtained from other diverse traffic data sources, and is a combined measure of page views and users. Alexa traffic Rank in Country is a rough estimate of the website popularity in a specific country. The global Alexa traffic rank and rank in Iran was extracted from the Alexa website on June 20, 2012 (22, 36).

### 3.6. Statistical Analysis

Correlations between variables were computed using non-parametric Spearman rho & Pearson correlation tests. We used Spearman and Pearson correlation analysis to explore associations between Bomba guidelines scores, total Bomba scores and global Alexa traffic rank, total Bomba scores and Alexa rank in Iran, Bomba scores and Google PageRank, Google PageRank and global Alexa traffic rank, Google PageRank and Alexa rank in Iran of the selected websites. Furthermore, the comparison between Site characteristic groups for each of the websites assessment tools (including, Alexa traffic global rank, Alexa traffic rank in Iran, Bomba total score and Google PageRank) was calculated by one-way between subjects ANOVA, Kruskal Wallis test, independent-samples t-test and Mann-Whitney's U test. The level of significance was set at 0.05.

The value of the coefficient ranges from -1 to 1. Values closer to +1 indicate a positive relationship, values closer to -1 indicate a negative relationship and values closer to 0 represent the absence of a relationship between the two variables. These analyses were performed using SPSS version 12.0.1. Table 3 is used to interpret correlation coefficients (37). The same interpretation also applies to negative correlations.

**Table 3.** Interpretation of Correlation Coefficient

Correlation Coefficient	Interpretation
**0.00-0.19**	slight, almost negligible correlation
**0.20-0.39**	low, quite small correlation
**0.40-0.69**	moderate correlation
**0.70-0.89**	high correlation
**0.90-1.00**	very high correlation

## 4. Results

### 4.1. Websites Characteristics

The characteristics of the 71 studied sites were summarized in Table 1. An independent-samples t-test was conducted to compare the Alexa traffic global rank, Alexa traffic rank in Iran and Bomba total score for MS SQL and My SQL Databases (Table 4). The difference was not statistically significant between the Alexa traffic global rank for MS SQL (M = 1244189. 38, SD = 1981420.75) and My SQL (M = 2029950.32, SD = 4824148.31) servers and also between the Alexa traffic rank in Iran for MS SQL (M = 10468.52, SD = 10352.65) and My SQL (M = 13251.47, SD = 16984.13) databases. In addition, there was no significant difference in the Bomba total score between the means of the two databases of MS SQL (M = 10. 14, SD = 1.60) and My SQL (M = 9. 90, SD = 1.82) databases. These results suggested that the database server type did not really have an effect on Alexa traffic global rank, Alexa traffic rank in Iran and Bomba total score.

The Mann-Whitney's U-test was conducted to evaluate the difference between the two database servers in Google PageRank. Database group had no significant effect on Google PageRank (The mean ranks of MS SQL and My SQL were 34.83 and 29.59, respectively; U = 411, N1 = 29, N2 = 34, Z = -1.19, P = 0.24). In this research, the independent samples t-test was used to compare the mean scores of two scope groups on the Alexa traffic global rank, Alexa traffic rank in Iran and Bomba total score (Table 4). Results indicated a non-significant preference on the Alexa traffic global rank for the general scope (M = 2302698.6, SD = 4893119.9) over the specific scope (M = 877063, SD = 895792.6). The 32 websites in the general scope (M = 12856.9, SD = 16995.7) and the 35 websites in the specific scope (M = 12368.3, SD = 13056) demonstrated a non-significant difference in Alexa traffic rank. There were no significant differences between general (M = 10.1, SD = 1.5) and specific (M = 9.8, SD = 1.8) scope in Bomba total score. Moreover, general and specific scope websites did not significantly differ in Google PageRank (Mann–Whitney U test: U = 526, N1 = 36, N2 = 35, Z = -1.25, P = 0.21).

**Table 4.** Independent-Samples T-test to Test Equality of Means of Database and Scope Categories for Alexa Traffic Global Rank, Alexa Traffic Rank in Iran and Bomba Total Score

	Database			Scope		
	t-test	df	Sig. (2-tailed)	t-test	df	Sig. (2-tailed)
**Alexa traffic global rank**						
Equal variances assumed	-0.82	61	0.42	-	-	-
Equal variances not assumed	-	-	-	1.7	69	0.09
**Alexa traffic rank in Iran**						
Equal variances assumed	-0.74	57	0.46	0.13	65	0.89
**Bomba final totalscore**						
Equal variances assumed	0.55	61	0.59	0.86	69	0.39

One-way ANOVA was conducted among subjects to compare the effect of the type of ownership structure on Alexa traffic rank in Iran and Bomba final total score in government, individual, and corporation conditions (homogeneity of variance was verified by using Levene's test). The analysis revealed a non-significant effect of the type of ownership structure on Alexa traffic rank in Iran [F (2, 62) = 1.03, p = 0.36] and Bomba final total score [F (2, 66) = 0.71, P = 0.50] at the significance level of 0.05 for the three conditions. The Kruskal Wallis test revealed a non-significant effect of ownership structure on Alexa traffic global rank (x^2^(2) = 4.78, P = 0.09) (homogeneity of variance was rejected by using Levene's test).

The Bomba score means and standard deviations for the total weighted value of six Bomba guidelines were presented in Table 5. As average, the highest Bomba scores (65%) was obtained from availability and usability guidelines.

**Table 5.** Scores of Bomba Guidelines

Guideline	Full Score	Mean (SD)	Minimum	Maximum
**Content**	6	1.7 (0.8)	0	3.6
**Usability**	5	3 (0.7)	1	4.8
**Availability**	4	3.5 (0.5)	2	4
**Advertising**	3	0.9 (0.6)	0	2.4
**Complimentary**	2	0.8 (0.5)	0	1.8
**Confidentiality**	1	0.1 (0.2)	0	1
**Final total score**	21	10 (1.7)	6.5	13.9

As Table 6 shows, most websites were in the moderate category (8 ≤ x ≤ 11.99) regarding their quality level (see Multimedia Appendix 1). The eight websites with good quality levels (12 ≤ x ≤ 15.99) based on the Bomba assessment included; 1) websites with general scopes: www.pezeshkonline.ir (total score = 13.9); www.hic.ir (total score = 12.5); Dr-ameri.com (total score = 12.20); www.myhealth.ir (total score = 12), and 2) websites with specific scopes: www.novindiet.com (score = 13.6); www.gabric.ir (score = 13.10); Mscenter.ir (score = 12.70); www.Charismaco.com (score = 12.60).

**Table 6.** Total Bomba Score and Quality Level for Each Category ^a^

Quality Level	Results
**Very poor**	0
**Poor**	7 (9.9)
**Moderate**	56 (78.9)
**Good**	8 (11.3)
**Excellent**	0
**Total**	71 (100)

^a^ Data are presented in NO. (%).

**Figure 2.**
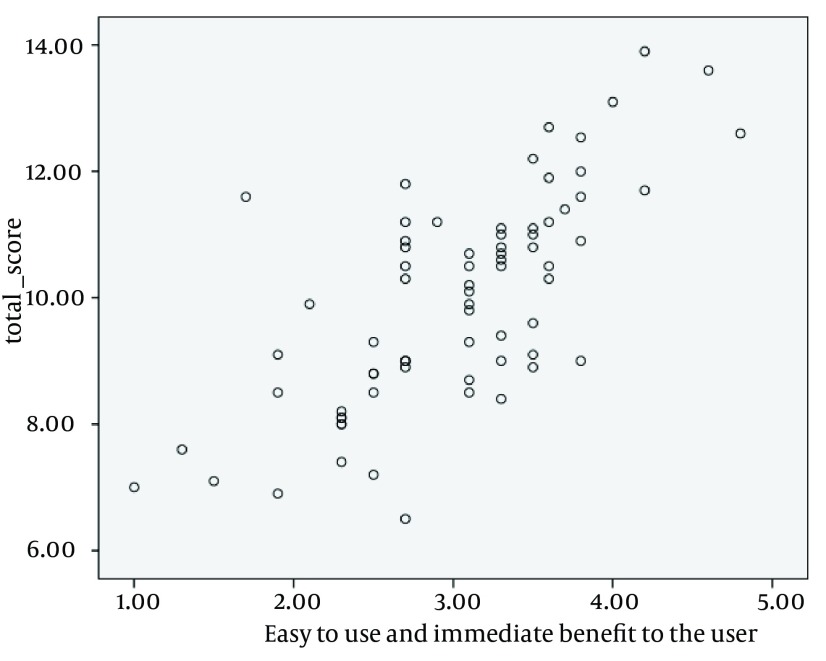
The Association Between Usability and Final Total Scores

**Figure 3.**
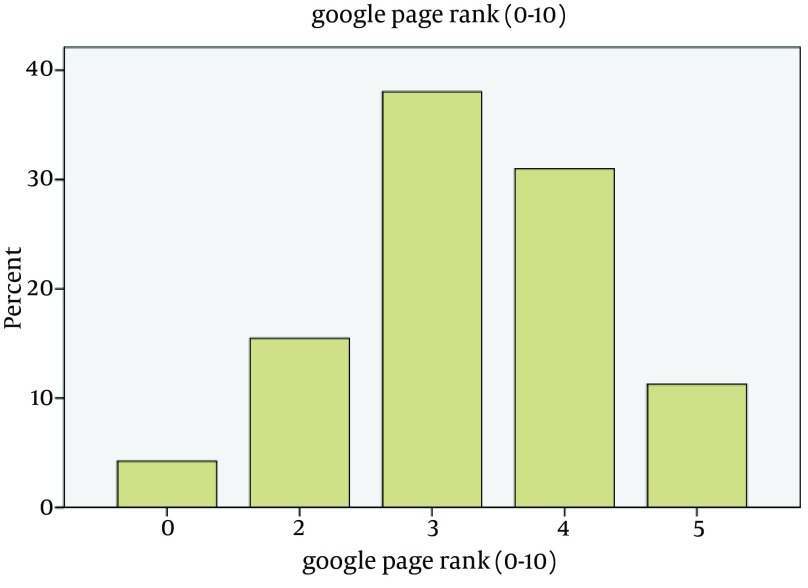
Percentage of the Websites in the Google Page Ranking Categories

Bivariate Pearson correlation was performed to evaluate correlation between different variables of Bomba index shown in Table 7. The Pearson correlation test revealed that five of six Bomba guidelines (content, usability, advertising, complimentary and confidentiality) had significant correlations with total scores. The highest correlation among the scores was found between usability and final total scores, demonstrating a significant and high correlation (r = 71, P < 0.001). Figure 2 shows this high correlation in a simple scatter graph. The scores of the two guidelines (complimentary and advertising) showed a low positive (0.20 ≤ r ≤ 0. 39) and statistically significant correlation (P < 0.001) with a larger number of the remaining guidelines (these two guidelines were correlated with all guidelines except for content and availability). There was a negative, low, and significant correlation between content and availability guidelines. It was shown that the more was the content score, the less was the availability score. Google PageRank scores were recorded for each of these 71 websites. There was no website with Google PageRank more than 5. Most websites (38%) had a PageRank of 3/10 (Figure 3).

**Table 7.** Pearson Correlation Between Bomba Guidelines, Bomba Guidelines and Final Total Score

	Content	Usability	Availability	Advertising	Complimentary	Confidentiality	Total Score
**Content**	1	0.07	-0.25 ^a^	-0.04	0.14	-0.08	0.44 ^a^
**Usability**	0.07	1	0.13	0.31 ^a^	0.32 ^a^	0.13	0.71 ^a^
**Availability**	-0.25 ^a^	0.13	1	-0.02	0.01	0.06	0.22
**Advertising**	-0.04	0.31 ^a^	-0.02	1	0.32 ^a^	0.27 ^a^	0.58^ a^
**Complimentary**	0.14	0.32 ^a^	0.01	0.32	1	0.31 ^a^	0.64 ^a^
**Confidentiality**	-0.08	0.13	0.06	0.27 ^a^	0.31*	1	0.35 ^a^
**Total Score**	0.44 ^a^	0.71 ^a^	0.22	0.58 ^a^	0.64*	0.35 ^a^	1

^a^ Correlation is significant at the 0.05 level (2-tailed).

The Alexa rank in Iran was available for 67 test sites as no regional data was available for the four websites. There were only 9 (12.75%) websites with global Alexa ranking below 100000 and 8 (11.9%) websites with Iran Alexa rank below 1000. Based on data obtained from Alexa, the “ninisite” website had the highest traffic rank in Iran (67) and in the world (4988) among others (Table 8).

**Table 8.** Descriptive Analysis of Google Page and Alexa Rank

Guideline	Mean (SD)	Minimum	Maximum
**Google PageRank (0-10)**	3.2 (1.1)	0	5
**Alexa traffic global rank**	1599920 (3588361)	4988	26851128
**Alexa traffic rank in Iran**	12602 (14951)	67	68845

**Figure 4.**
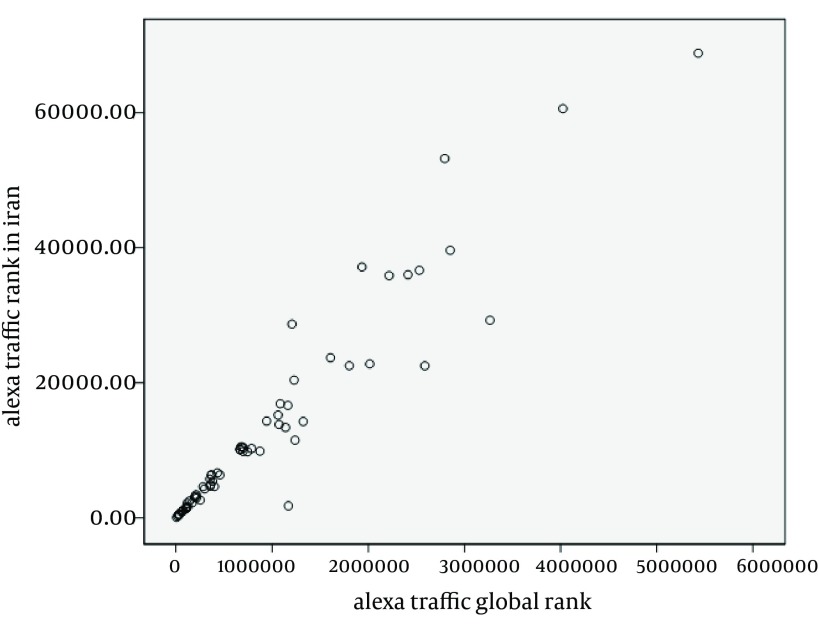
Correlation Between Alexa Traffic in the World and Iran

The study of the association between the Google PageRank, Bomba index variables (guideline scores and total score), Alexa traffic rank in Iran and Alexa traffic global rank showed some valuable information illustrated in Table 9. Spearman's correlation coefficients between Google PageRank with Bomba index variables and Alexa traffic global rank showed no statistically significant association between them (P > 0.05). However, the association between the PageRank and Alexa traffic rank in Iran revealed a statistically low negative correlation coefficient (rho = -0.29, P = 0.02). Results of Pearson's test indicated a low negative significant correlation between the Alexa rank in Iran with complimentary guideline (r = -0.31, P = 0.01), and Alexa rank in Iran with Bomba total score (r = -0.29, P = 0.02). Similarly, the Alexa traffic global rank was low, negatively correlated with the complimentary score (r = -0.25, P = 0.04). However, a moderate but statistically significant negative correlation was detected between the advertising guideline and Alexa traffic rank in Iran (r = 0.44, P = 0.00). As Figure 4 shows, the strongest significant positive correlation was found between Alexa traffic rank in Iran and Alexa traffic rank in the world (r = 0.96, P = 0.00). By contrast, there was no significant correlation between other variables (P > 0.05).

**Table 9.** Correlation Between Bomba Guidelines Scores With Google PageRank

	Google Page Rank	Alexa Rank in Iran	Alexa Traffic global rank	Bomba Index						
				Content	Usability	Availability	Advertising	Complimentary	Confidentiality	Final total Score
**Google PageRank (spearman's rho)**	1	-0.29 ^a^	0.22	0.21	-0.22	-0.10	0.04	-0.10	-0.15	-0.09
**Alexa traffic rank in Iran (Pearson Correlation)**	-0.29 ^a^	1	0.96 ^a^	-0.13	-0.05	0.12	-0.44 ^a^	-0.31 ^a^	0.04	-0.29 ^a^
**Alexa traffic global rank (Pearson Correlation)**	0.22	0.96 ^a^	1	-0.12	0.11	0.13	-0.03	-0.25 ^a^	-0.09	-0.06

^a^ Correlation is significant at the 0.05 level (2-tailed).

## 5. Discussion

### 5.1. Principal Results and Comparison With Prior Works

To our knowledge, this is the first quality assessment of Persian language health website, which also evaluated the correlation between popularity and importance rank with the quality score on the Internet. Study of the characteristics of selected websites showed that most websites were developed by persons with the objective of health promotion, maybe due to increased interest to health related subjects and computer knowledge of the Iranian people. In addition, most sites were established with the aim of promoting health. However, site ownership was distributed relatively evenly between individuals and organizations in another study aimed to determine website quality indicators for consumers (38). Furthermore, Vakili evaluated online health information about hepatitis B and found that most infectious diseases websites were developed by organizations to provide education and information services (39). Nevertheless, the disease was the main focus of websites in a previous evaluation of Persian medical and health websites using Silberg criteria (19). These contradictory findings are due to transformation in attitude toward health promotion and prevention. There was no significant difference in the Alexa traffic global rank, Iran Alexa traffic rank and Bomba total score between the site groups based on their characteristics of ownership structure, database, scope and objective, and none of these specifications had really an impact on the quality score and websites ranks . However, evaluation of websites providing information about urinary incontinence by using general quality criteria based on Silberg et al. and the HONcode principles showed that the difference between organizations and commercial sites was significant as it was between professional and commercial sites (40). The differences in the results might be due to the differences in studied websites, assessment instruments and scoring systems.

The total Bomba score of the most of the test websites puts them in the moderate category based on the predefined quality level. The websites received better quality scores in availability and usability guidelines as compared with the other guidelines. However, the operationalised subitems of the content guideline were not considered by the websites owners, in spite of having the highest rank (rank 1) and weighted multiplier (6) in the Bomba scoring system. The weakness in content and non-compliance with the standards of quality-assessment instruments were also indicated in other studies (3, 5, 8, 20, 25, 41, 42). Moreover, there was a negative, low, and significant correlation between content and availability guidelines; therefore, availability subitems such as the URL accessibility, home page load time, link checking and browser compatibility should be considered in addition to the procedures performed for content improvement. None of the top Persian medical and health websites identified in the current study by the Bomba rating system were similar to those specified by the Silberg ranking system (19). This difference might be caused by the weighted multiplier, ranking the guidelines and special scoring system used in Bomba index that provided the possibility of more accurate ranking and comparison of websites. Two scores of two guidelines (complimentary and advertising) of Bomba showed a positive and statistically significant correlation (P < 0.05) with a larger number of remaining guidelines. Assessing the quality of websites providing health-related information for patients in Spanish showed a statistically significant correlation between two dimensions of editorial policy and accessibility in compliance with codes of conduct with a larger number of remaining factors (43). The usability guideline score had the most effect on total Bomba scores. Hence, the more the usability score, the more the total score in most cases. This high correlation can be due to the high weighted multiplier of this guideline (= 5) and poor quality of content guidelines in selected websites. Therefore, the usability guideline score was predictive of the final total Bomba score of these websites.

Most websites had a Google PageRank of 3/10 on their home pages. It means that they were in average position and had reasonable authority. The lowest and highest identified Page Ranks of assessed website were 0 and 5, respectively although the PR7 websites were seen in assessment of the quality of depression websites by Griffiths et al. (10). The PageRank only had a weak correlation with Alexa traffic rank in Iran. No significant association existed between PageRank and Bomba quality scores (guideline scores and total score). Therefore, PageRank does not properly reflect the real quality of websites and internet users seeking online health information should not merely rely on it for any kind of prejudgment regarding Persian health websites. Similarly, there was an insignificant correlation between Google PageRank and the evidence-based score in a study conducted by Griffiths et al. on depression websites. In contrast, regarding other previous studies in this field, there was a correlation between Google PageRank with evidence-based quality score, consumer/ expert satisfaction and Web Accessibility Barriers (WAB) score (36, 38, 44, 45).

A minority of test websites had a good Alexa ranking. The unsuccessful performance of websites of Iranian medical universities regarding the Alexa rank was investigated in another study (46). Moreover, a significant correlation was found between Iran Alexa ranking and guidelines of complimentary and advertising and as well as Bomba total score. Therefore, it can be concluded that care about the sub items of these guidelines such as contact information of health professional, online consultation, various levels of information, feasibility of asking questions and expressing opinions, editorial policy and transparency of authorship and sponsorship can increase the popularity of the studied websites. Iran Alexa rank is a relatively promising automatic indicator of the website quality; hence, it can be used as a primary filtering tool by visitors of the Persian health websites. However, other studies showed no correlation between websites popularity indices with quality scores and WAB (36, 38, 40, 42). A very high correlation was found between Alexa traffic rank in Iran and the world, which could be due to the great percentage of visitors for the selected websites from Iran (86.6%). Finally, based on the obtained results and the significance of online health information, the following recommendations are proposed to have high-quality Persian health websites:

Assigning organizations for formal quality evaluation of health websites;

Designing a search engine dedicated to explore accredited health related websites;

Labeling of qualified health websites;

Establishing quality standards of health websites;

Exploring an automated tool for quality evaluation of online health information;

Educating site developers about a set of quality criteria for health websites.

There were several limitations in this study. First, some websites were removed from the sample because of filtering and technical problems of the websites. Second, Alexa traffic data is obtained via information of the Alexa toolbar users, which is only available for the Firefox and Internet Explorer on Windows operating systems. Therefore, Alexa is not able to track the traffic of the websites through other browsers or operating systems. Third, website developers may use some methods to artificially inflate the Alexa ranking and Google PageRank without any changes in the content of their websites. Finally, the Internet is a constantly changing environment. Therefore, it is impossible to represent a continual picture of the studied websites and the results of this study would be changed in the course of time.

**Appendices**

## Appendices

**Appendix 1.** Bomba and Land Index (version 2)

Websites	Traffic Rank		Google Page Rank	Bomba Index						
	Global	In Iran		Content	Usability	Availability	Advertising	Complimentary	Confidentiality	Total Score
**ninisite.com**	4988	67	4	1.8	3.1	3.5	1.2	0.8	0.3	10.7
**www.iransalamat.com**	17623	263	4	2.4	2.5	3.0	0.3	1.0	0.1	9.3
**www.parsiteb.com**	27592	465	3	1.2	3.6	4.0	1.5	0.6	0.3	11.2
**iec.behdasht.gov.ir**	29687	447	5	1.5	2.1	4.0	1.5	0.8	0.0	9.9
**www.pezeshk.us**	32436	461	4	2.1	2.7	3.5	1.2	1.0	0.0	10.5
**www.salamatnews.com**	35662	564	5	2.4	2.5	2.5	0.9	0.2	0.0	8.5
**www.pezeshkan.org**	57586	893	4	0.9	2.5	3.5	1.2	0.7	0.0	8.8
**www.irteb.com**	64617	994	4	1.8	2.7	3.5	1.8	1.0	0.1	10.9
**www.irshafa.ir**	72956	1099	3	2.4	3.3	4.0	0.6	0.5	0.0	10.8
**tanzimekhanevadeh.com**	101380	1331	3	1.2	3.3	3.0	1.8	1.0	0.3	10.6
**www.salamatiran.com; www.salamatiran.org**	104671	1460	5	2.4	3.1	3.0	1.2	0.2	0.0	9.9
**www.pezeshkonline.ir**	106797	1576	3	3.0	4.2	3.5	1.2	1.7	0.3	13.9
**www.hic.ir; www.iranhealers.com**	109798	1481	4	2.4	3.8	4.0	1.2	1.0	0.1	12.5
**www.ravazadeh.com**	115745	1728	3	0.9	3.8	3.5	1.8	0.6	1.0	11.6
**3-m.ir**	116111	1551	3	3.0	2.9	2.5	1.5	1.0	0.3	11.2
**www.dr-ameri.com**	116776	2146	3	2.4	3.5	4.0	0.9	1.3	0.1	12.2
**www.novindiet.com**	138050	2509	3	1.8	4.6	4.0	1.5	1.3	0.4	13.6
**www.pezeshki.net**	169110	2244	4	1.8	3.3	3.0	1.2	1.3	0.1	10.7
**worldfood.ir**	193076	3066	3	1.8	2.7	3.5	0.6	0.3	0.0	8.9
**www.drzohrabi.ir**	204202	3206	3	2.7	2.7	3.5	0.6	1.3	0.0	10.8
**www.irden.com**	209186	3445	4	1.8	1.7	4.0	1.8	1.5	0.8	11.6
**www.unknown.ir**	211206	2934	3	0.3	2.3	4.0	0.9	0.2	0.3	8.0
**mscenter.ir**	252481	2645	2	2.4	3.6	3.5	1.2	1.3	0.7	12.7
**www.sportmedicine.ir**	279977	4629	3	2.4	3.5	3.0	1.2	0.6	0.1	10.8
**www.myhealth.ir**	296560	4287	3	3.6	3.8	2.0	1.2	1.3	0.1	12.0
**isaarsci.ir**	352770	5762	3	2.4	2.7	3.5	0.7	1.0	0.0	10.3
**www.darooyab.ir**	353827	4718	4	1.8	2.7	3.0	1.2	0.3	0.0	9.0
**salemzi.com**	358435	4928	3	1.8	2.5	4.0	0.3	0.2	0.0	8.8
**www.iranhiv.com**	365177	6309	5	0.6	3.5	4.0	0.0	0.8	0.0	8.9
**hamdardi.com**	367183	6375	3	0.9	2.7	3.0	1.2	0.6	0.6	9.0
**www.salamatestan.com**	380692	5405	2	2.4	3.5	3.5	0.9	0.8	0.0	11.1
**www.behsite.ir**	399104	4658	4	1.8	3.6	3.0	0.6	1.3	0.0	10.3
**www.boali.com**	429214	6677	4	1.8	1.9	3.5	0.6	1.3	0.0	9.1
**www.congress60.org**	457246	6355	4	0.9	3.7	4.0	1.2	1.3	0.3	11.4
**dr.avazeh.ir**	662418	10169	2	0.6	1.9	3.5	0.6	0.3	0.0	6.9
**www.moshavercard.com**	667100	10120	2	0.6	3.5	3.5	2.4	1.0	0.0	11.0
**parsdiet.com**	675338	10512	4	1.5	1.9	3.5	0.6	1.0	0.0	8.5
**www.salamat.ir**	689274	10503	5	2.4	3.1	3.5	0.9	0.2	0.0	10.1
**www.iranblood.org**	696997	9850	3	2.7	1.3	2.5	0.3	0.7	0.1	7.6
**www.gabric.ir**	701784	10375	3	2.4	4.0	4.0	1.5	1.2	0.0	13.1
**www.orzhans.ir**	743515	9804	3	0.0	3.3	3.5	2.1	1.8	0.3	11.0
**www.irandentist.info**	785164	10271	4	1.0	2.3	3.0	0.6	0.5	0.0	7.4
**www.rejimdarmani.com**	872108	9901	3	2.4	2.7	4.0	1.2	1.5	0.0	11.8
**labnews.ir**	941524	14345	2	0.9	2.3	3.0	0.3	1.3	0.4	8.2
**charismaco.com**	1061503	15238	3	0.9	4.8	3.5	1.8	1.3	0.3	12.6
**lifestyle.timclinic.ir**	1071187	13840	2	0.0	2.7	3.5	0.0	0.3	0.0	6.5
**geneclinic.ir**	1083090	16909	3	1.2	3.3	3.0	0.3	0.5	0.1	8.4
**www.imeny.com**	1138677	13392	4	1.5	3.1	3.5	1.5	0.8	0.1	10.5
**www.taamasrar.com**	1162980	16654	4	0.6	3.8	3.5	0.6	0.5	0.0	9.0
**www.iranderma.com**	1167785	1792	4	1.2	1.0	4.0	0.3	0.5	0.0	7.0
**www.msworld.ir**	1205917	28718	2	0.6	3.1	3.5	0.0	1.7	0.4	9.3
**www.shafagar.com/farsi**	1227128	20424	2	0.3	2.5	3.5	0.9	0.0	0.0	7.2
**www.medapple.com**	1237119	11505	2	2.4	3.6	3.5	0.0	1.0	0.0	10.5
**healthtube.ir**	1322626	14281	5	2.4	3.1	3.5	0.6	0.6	0.0	10.2
**irhiv.com**	1606195	23728	2	0.9	3.3	4.0	0.6	0.2	0.0	9.0
**radiosalamat.ir**	1801364	22548	5	1.5	3.5	3.5	0.3	0.7	0.1	9.6
**www.pishgirinovin.com**	1933175	37174	4	1.8	2.3	4.0	0.0	0.0	0.0	8.1
**iranems.com**	2014478	22803	4	1.8	2.3	3.5	0.0	0.5	0.0	8.1
**www.samas.ir = moshaver.behdasht.gov.ir**	2216232	35895	4	2.4	2.7	4.0	0.3	0.8	1.0	11.2
**www.bavariran.com**	2412465	36021	3	1.8	3.8	3.5	1.2	0.6	0.0	10.9
**irmna.com**	2529673	36685	3	1.2	3.1	3.5	0.6	0.3	0.0	8.7
**www.drpalapal.com**	2587050	22533	0	1.2	4.2	4.0	0.6	1.7	0.0	11.7
**medlineir.com**	2794663	53241	0	1.2	1.5	3.5	0.9	0.0	0.0	7.1
**www.medtube.ir**	2851700	39637	5	2.4	2.3	2.5	0.3	0.5	0.0	8.0
**www.khornoush.com**	3264482	29286	2	1.8	3.3	4.0	0.0	0.3	0.0	9.4
**www.iranradiology.ir**	4023699	60635	3	1.8	3.1	3.5	0.0	0.0	0.1	8.5
**www.iranonlinedoctor.com**	5430151	68845	0	1.8	3.3	4.0	0.3	0.8	0.3	10.5
**www.npjm.org**	5735929	null	4	2.40	3.10	3.50	0.6	0.2	0.0	9.8
**www.sepidweekly.ir**	9536523	null	4	2.40	3.30	4.00	1.2	0.2	0.0	11.1
**www.tabehesht.com**	10360292	null	3	1.8	3.6	4.0	1.5	1.0	0.0	11.9
**salamatniaz.com**	26851128	null	3	0.6	3.50	3.5	1.2	0.2	0.1	9.1
